# Heart Rate and Heart Rate Variability Are Affected by Age and Activity Level in Athletic Horses

**DOI:** 10.3390/vetsci12070624

**Published:** 2025-06-28

**Authors:** Thita Wonghanchao, Kanokpan Sanigavatee, Soontaree Petchdee, Kulpreeya Chettaratanont, Thitakorn Thongyen, Boonbaramee Wanichayanon, Chanoknun Poochipakorn, Metha Chanda

**Affiliations:** 1 Department of Large Animal and Wildlife Clinical Science, Faculty of Veterinary Medicine, Kasetsart University, Kamphaeng Saen Campus, Nakorn Pathom 73140, Thailand; thita.wo@ku.th (T.W.); ksanigavatee@gmail.com (K.S.); fvetstr@ku.ac.th (S.P.); 2 Veterinary Science Program, Faculty of Veterinary Medicine, Kasetsart University, Kamphaeng Saen Campus, Nakorn Pathom 73140, Thailand; kulpreeya.c@ku.th (K.C.); thitakorn.t@ku.th (T.T.); boonbaramee.w@ku.th (B.W.)

**Keywords:** autonomic regulation, dressage, exercise volume, geriatric, horse, school riding, training, vagal, sympathetic, welfare

## Abstract

The physiological capabilities of horses begin to decline after they reach 15 years of age. However, some exceptionally talented horses can continue their sports careers beyond this age, raising concerns about their welfare. This study investigated animal welfare by observing resting heart rate (HR) and heart rate variability (HRV) metrics in geriatric horses participating in similar training or physical activities compared to younger adult horses over 30 min periods. Forty-six horses of varying ages and activity levels were divided into four groups, including horses under 15 years practising structured training for dressage (AL-1; N = 13) and school riding (AL-2; N = 13) and horses aged between 15 and 20 years participating in AL-1 (N = 10) and AL-2 (N = 10). In adult horses, the HR decreased in those performing AL-1, which was observed alongside an increased RR interval compared to those participating in AL-2 (*p* < 0.05 for both). Meanwhile, HRV increased in adult horses (measured by pNN50) but decreased in geriatric horses (measured by SDANN, SDNNI, DC, and SD2) participating in AL-1 when compared to their counterparts in AL-2 (*p* < 0.05 for all). Adult horses exhibited better autonomic regulation while engaged in structured dressage training than geriatric horses. These findings underscore the necessity for the careful management of physical activities to ensure the well-being of all horses, particularly older ones.

## Simple Summary

Horses are utilised for various purposes, particularly within the equestrian industry. Typically, a horse’s physiological functions begin to decline after they reach 15 years of age. However, exceptional performers or those with specialised skills may continue to compete beyond this age, which raises welfare concerns for older horses. This study evaluates animal welfare by measuring resting heart rate (HR) and heart rate variability (HRV) in four groups of horses with different ages and activity levels. Activity level, as an independent factor, and its interaction with age significantly influenced HR and multiple HRV metrics. Horses under 15 years of age exhibited a decreased HR and increased HRV when engaged in structured exercise for dressage training, suggesting a shift toward vagal dominance compared to horses involved in school riding. In contrast, horses aged 15 to 20 years showed lower HRV during structured dressage training, indicating reduced vagal tone compared to their counterparts in school riding. Therefore, caution is necessary when continuing structured training for equestrian sports in horses older than 15 years. These findings have important implications for horse welfare, highlighting the need for careful management and appropriate physical activities to safeguard the well-being of older horses.

## 1. Introduction

Globally, equestrian sports are growing in popularity at national and international levels. The Fédération Equestre Internationale (FEI) Database lists approximately 478,000 horses involved in equestrian sports, with nearly 77,000 horses registered by national federations each year [1]. The increasing number of horses involved in the equestrian industry coincides with growing concerns regarding animal welfare, particularly for older horses [2]. Ageing refers to the decline over time in the functioning of vital organs, including the cardiovascular system [3,4], which can lead to reduced exercise capacity and impaired thermoregulation [3]. Additionally, the maximal rate of oxygen consumption (VO_2max_) in horses tends to decrease with age [4]. Research indicates that older horses also exhibit reduced muscle oxidative capacity and a shift towards more glycolytic properties [5]. Thus, ageing negatively impacts bodily systems and potentially impairs homeostasis. It is widely accepted that physiological functions in horses start to decline after they reach 15 years of age, marking the onset of senescence [6,7]. However, some horses continue to compete successfully even beyond the age of 20 [7,8]. The use of older horses in equestrian sports—when their physical abilities are naturally diminished—may expose them to increased stress and could negatively affect their well-being during training or competition.

Assessing physiological stress parameters at rest or during groundwork can provide valuable insights into the effects of stress levels and the adaptations that occur following exercise training [9,10,11,12]. In this regard, heart rate (HR) and heart rate variability (HRV) have frequently been used to monitor the stress and neurophysiological responses to various conditions and challenges in both humans [13,14,15,16] and horses [9,17,18]. HRV describes a rhythmic fluctuation in time intervals between consecutive heartbeats. This variation is mediated by sympathetic and parasympathetic (vagal) impulses acting on the sinoatrial node of the heart during the cardiac cycle [19,20,21,22]. This natural phenomenon reflects the body’s flexibility and adaptability to cope with stressful stimuli and maintain homeostasis [20,23].

Several HRV metrics can indicate the degree of independent vagal influence or the combined activity of sympathetic and vagal systems. These metrics can be analysed through three approaches, namely time-domain, frequency-domain, and nonlinear methods. Changes in time-domain variables, such as the beat-to-beat (RR) interval, the standard deviation of normal-to-normal RR intervals (SDNN), the standard deviation of the averages of RR intervals over 5 min segments (SDANN), and the mean of the standard deviations of RR intervals in 5 min segments (SDNNI), reflect long-term variation and are influenced by the interplay between sympathetic and vagal components. Additionally, modulation of the frequency-domain variable, specifically the low-frequency (LF) band, and nonlinear variables, such as the standard deviation of the Poincaré plot along the line of identity (SD2), are affected by both sympathetic and vagal components [21,24]. Conversely, an increase in certain time-domain variables, such as the square root of the mean squared differences between successive RR intervals (RMSSD), the high-frequency (HF) band in frequency-domain metrics, and nonlinear variables like the standard deviation of the Poincaré plot perpendicular to the line of identity (SD1), indicates short-term variations that signify vagal dominance [20,21]. A reduction in HRV reflects compromised autonomic regulation, which may negatively impact horse welfare [25]. A decrease in resting HR and multiple HRV metrics has been reported in horses as they age [26,27]. However, these physiological responses can be improved in geriatric horses through structured exercise [12,28].

Although geriatric horses can be trained for and succeed in equestrian competitions, it is still unclear to what extent their welfare is maintained when their sports careers extend beyond the age at which their physiological functions begin to decline. This study aimed to investigate animal welfare by observing resting HR and various HRV metrics in geriatric horses involved in equestrian sports or physical training. These geriatric horses were compared to adult horses under similar conditions. The hypothesis was that resting HR and HRV metrics would differ between adult and geriatric horses engaged in various levels of activity. Additionally, it was expected that both age and activity levels would influence HR and HRV metrics.

## 2. Materials and Methods

### 2.1. Horses

Fifty-one mixed-native horses (4 stallions, 24 geldings, 23 mares) aged between 7 and 20 years and weighing 321–435 kg were selected from the Horseshoe Point Riding Club, Chonburi, Thailand (location: 12.906695, 100.96976). The horses participated in various physical activities, including regular training for equestrian sports—particularly dressage—as well as school riding and rest periods since they were six. The horses were housed in well-ventilated 3 × 4 m stables with straw bedding, with ad libitum tap water and pangola hay. Approximately 1.5–2 kg of commercial pellets with 90 g of electrolytes were given daily across three distribution times. On non-exercise days, the horses were allowed to graze in the paddock for 2 to 3 h. Before the study began, all horses underwent physical and echocardiographic examinations conducted by a specialist veterinarian to identify any cardiac or musculoskeletal disorders. No horses were found to have musculoskeletal problems or lameness; however, five were excluded from the study due to cardiac arrhythmia, manifesting as bradycardia, atrioventricular block, and supraventricular premature beats. Accordingly, data were collected for 46 horses in this study. The animal study protocol was approved by the Institutional Review Board (or Ethics Committee) of the Kasetsart University Institute of Animal Care and Use Committee (ACKU68-VET-023; 1 April 2025).

### 2.2. Experimental Protocols

Horses were categorised into four groups based on their ages and activity levels, as shown in Table 1. The activity levels fell into the two following patterns: (1) Horses engaged in daily aerobic training, especially for equestrian dressage, which included 15–20 min of walking, 15–20 min of trotting, and 10 min of cantering. The total duration was approximately 40–50 min per day, three to five days a week (AL-1). (2) Horses were used for school riding by practising combined walking and trotting for 35–45 min, along with a 5 min canter, one day a week or less (AL-2). Horses were left in the paddock for two to three hours on non-exercise days.

Horses were equipped with a heart rate monitoring (HRM) device (Polar Electro Oy, Oulu, Finland) while they were individually placed in front of their home stables to determine the resting HR and HRV. This HRM device has been demonstrated to generate reliable RR interval data from which HRV metrics can be derived [29,30,31,32]. A Polar equine belt for riding was soaked in water and then fitted, using ultrasound gel at the electrode surface to enhance electrical transmission. A heart rate sensor (H10) was subsequently attached to the belt and fastened around the chest, with the sensor pocket positioned on the left side of the chest. Finally, the sensor was wirelessly linked to a Polar sports watch (Vantage 3) to obtain RR interval data. The HRM device was installed on the horses for approximately 10 min before each 30 min session of data recording. This acclimatisation process helps familiarise the horses with the device, minimising potential anxiety-related distortions in HRV metrics. Data were recorded on each horse’s non-exercise days for two consecutive days between 08:00 and 16:00 to avoid any influence of physical activity on HRV metrics.

The Polar sports watch was connected to the FlowSync software programme version 4 (https://flow.polar.com/start, accessed on 13 March 2025) to transfer RR interval data, which was subsequently exported as comma-separated value (CSV) files. These files were then uploaded to the Kubios premium programme version 4.1.2.1 (Kubios HRV scientific: https://www.kubios.com/hrv-premium/, accessed on 13 March 2025) for the calculation of specific HRV metrics. HRV data were eventually exported as MATLAB MAT files. HRV measurements can be distorted by artefacts such as missing, extra, misaligned, or ectopic beats (including premature ventricular contractions or other arrhythmias). To address this issue, an automatic artefact correction feature was utilised to ensure the accuracy of the aligned RR intervals. Additionally, automatic noise detection was applied at a medium level to filter out noisy segments that could lead to distortions in the analysis of consecutive beats. The smoothness prior method was also implemented to eliminate non-stationarities within the RR interval time series. The cutoff frequency set for trend removal was 0.035 Hz, as denoted in the user guidelines (https://www.kubios.com/downloads/Kubios_HRV_Users_Guide.pdf, accessed 13 March 2025).

HRV metrics derived from 30 min recordings were reported in three domain analyses, including (1) a *time-domain analysis*: mean HR, RR interval, SDNN, SDANN, SDNNI, RMSSD, pNN50, and deceleration capacity of heart rate computed as a four-point difference (DC); (2) *frequency-domain analysis*: LF band (frequency threshold: 0.01–0.07 Hz), HF band (frequency threshold: 0.07–0.6 Hz), total power, and LF/HF ratio; and (3) *nonlinear analysis*: SD1, SD2, and the SD2/SD1 ratio.

### 2.3. Data Analysis

HRV data were statistically analysed using GraphPad Prism version 10.4.2 (GraphPad Software Inc., San Diego, CA, USA). A two-way repeated measures ANOVA was utilised to determine the independent effects of horses’ age and activity levels, as well as the interaction effect between horses’ age and activity levels on the observed HR and HRV metrics. Differences within and between groups were evaluated using uncorrected Fisher’s least significant difference (LSD) test. The effect size was determined using Eta squared (*η*^2^) and denoted as very small (*η*^2^ < 0.01), small (0.01 ≤ *η*^2^ < 0.06), medium (0.06 ≤ *η*^2^ < 0.14), or large (*η*^2^ ≥ 0.14) [33]. The results are presented as mean ± SD, and statistical significance was considered at *p* < 0.05.

## 3. Results

### 3.1. Time-Domain Results

An independent effect for activity levels was found on the expression of the mean HR (F_(1, 21)_ = 5.376, *p* = 0.0306, *η*^2^ = 0.10), mean RR (F_(1, 21)_ = 4.643, *p* = 0.0429, *η*^2^ = 0.08), SDANN (F_(1, 19)_ = 8.158, *p* = 0.0101, *η*^2^ = 0.15), and SDNNI (F_(1, 19)_ = 4.947, *p* = 0.0385, *η*^2^ = 0.12). Meanwhile, the interaction between age and activity level influenced the expression of pNN50 (F_(1, 21)_ = 5.714, *p* = 0.0263, *η*^2^ = 0.09), DC (F_(1, 21)_ = 4.460, *p* = 0.0468, *η*^2^ = 0.09), and the stress index (F_(1, 21)_ = 5.985, *p* = 0.0233, *η*^2^ = 0.10). Neither independent nor interaction effects influenced RMSSD and SDNN measurements.

**Figure 1:**
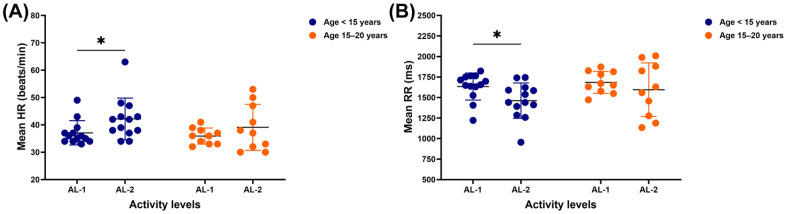
Mean heart rate (**A**) and mean RR interval (**B**) in horses across different ages and activity levels. * indicates significant difference at *p* < 0.05. HR: heart rate; RR: beat-to-beat; AL-1: structured dressage training; AL-2: school riding practice.

In horses under 15 years of age, the mean HR was lower for those in the AL-1 group than those in AL-2 (37.1 ± 4.4 vs. 42.2 ± 7.6 beats/min, *p* = 0.0428). However, no mean HR difference was observed in horses between the ages of 15 and 20 years when performing AL-1 and AL-2 (Figure 1A). Meanwhile, the mean RR was higher in horses younger than 15 years practising AL-1 compared to those practising AL-2 (1633.9 ± 165.2 vs. 1461.8 ± 215.5 ms, *p* = 0.0434). There was no variation in the mean RR in horses aged 15–20 years between the AL-1 and AL-2 groups (Figure 1B).

**Figure 2:**
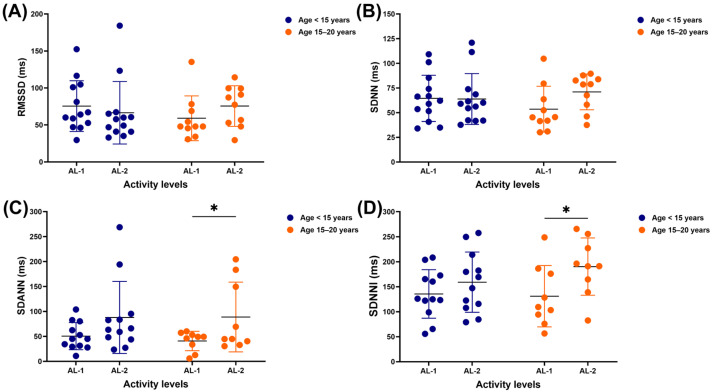
HRV metrics: RMSSD (**A**), SDNN (**B**), SDANN (**C**), and SDNNI (**D**) in horses across different ages and activity levels. * indicates significant difference at *p* < 0.05. HRV: heart rate variability; RR: beat-to-beat; RMSSD: square root of the mean squared differences between successive RR intervals; SDNN: standard deviation of normal-to-normal RR intervals; SDANN: standard deviation of the averages of RR intervals in 5 min segments; SDNNI: mean of the standard deviations of RR intervals in 5 min segments; AL-1: structured dressage training; AL-2: school riding practice.

No significant differences were detected in the RMSSD and SDNN of horses in either age range when performing AL-1 and AL-2 (Figure 2A,B). SDANN and SDNNI did not differ between AL-1 and AL-2 in horses aged less than 15 years; however, in older horses, both were lower in those participating in AL-1 than those participating in AL-2 (SDANN: 40.8 ± 19.5 vs. 88.9 ± 70.0 ms, *p* = 0.0471; SDNNI: 131.0 ± 61.4 vs. 190.3 ± 57.3 ms, *p* = 0.0487) (Figure 2C,D).

**Figure 3:**
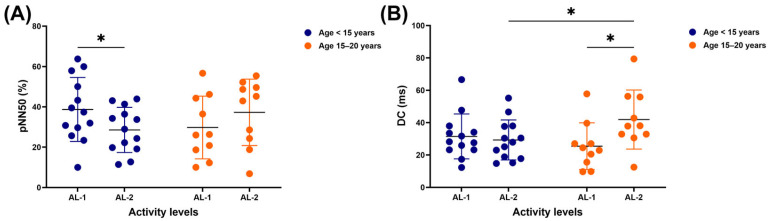
HRV metrics: pNN50 (**A**) and DC (**B**) in horses across different ages and activity levels. * indicates significant difference at *p* < 0.05. HRV: heart rate variability; RR: beat-to-beat, pNN50: number of successive RR interval pairs that differ more than 50 ms divided by the total number of RR intervals; DC: deceleration capacity of heart rate computed as a four-point difference; AL-1: structured dressage training; AL-2: school riding practice.

A significant difference in pNN50 was observed only in horses under 15 years old, with higher values for those practising AL-1 compared to those practising AL-2 (38.7 ± 15.9 vs. 28.6 ± 11.2 %, *p* = 0.0494) (Figure 3A). Although there was no significant difference in DC between AL-1 and AL-2 in younger horses, a higher value was observed in horses 15–20 years old who participated in AL-2 compared to those performing AL-1 (41.9 ± 18.2 vs. 25.5 ± 14.4 ms, *p* = 0.0216) and younger horses performing AL-2 (41.9 ± 18.2 vs. 29.3 ± 12.3 ms, *p* = 0.0469) (Figure 3B).

### 3.2. Frequency-Domain Results

**Figure 4:**
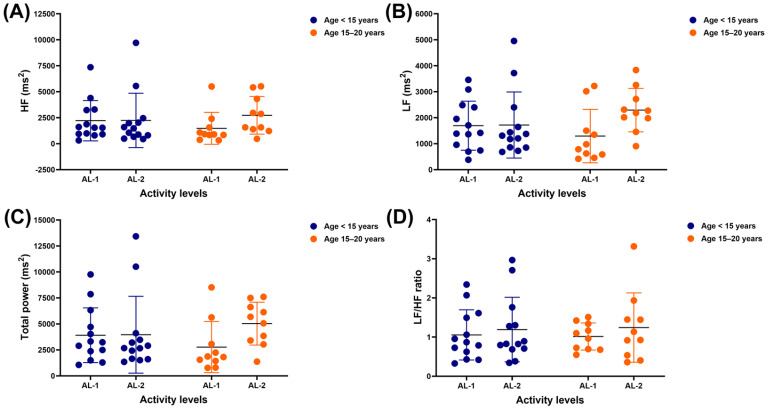
HRV metrics: HF band (**A**), LF band (**B**), total power band (**C**), and LF/HF ratio (**D**) in horses across different ages and activity levels. HRV: heart rate variability; HF: high frequency; LF: low frequency; AL-1: structured dressage training; AL-2: school riding practice.

Neither independent nor interaction effects were observed in the HF, LF or total band power, nor the LF/HF ratio. While there were no significant differences in the frequency-domain results, a non-significant difference was observed between AL-1 and AL-2 in the LF band (*p* = 0.0513) and total power (*p* = 0.0969) (Figure 4A–D).

### 3.3. Nonlinear Results

**Figure 5:**
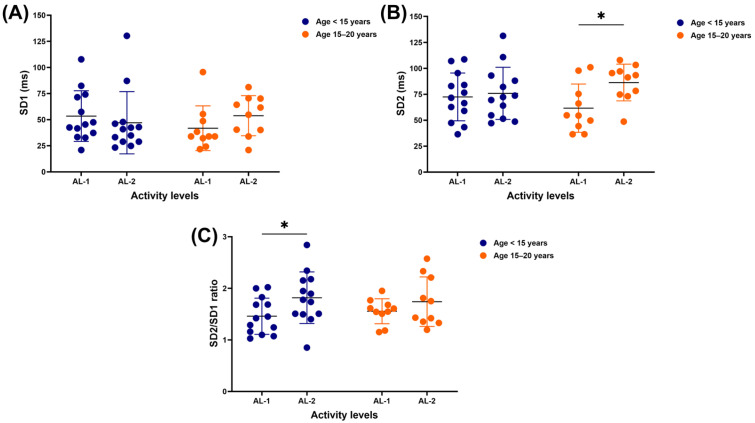
HRV metrics: SD1 (**A**), SD2 (**B**), and SD2/SD1 ratio (**C**) in horses across different ages and activity levels. * indicates significant difference at *p* < 0.05. HRV: heart rate variability; SD1: standard deviation of Poincaré plot perpendicular to the line of identity; SD2: standard deviation of Poincaré plot along the line of identity; AL-1: structured dressage training; AL-2: school riding practice.

Activity level independently impacted the expression of SD2 (F_(1, 21)_ = 4.353, *p* = 0.0493, *η*^2^ = 0.09) and the SD2/SD1 ratio (F_(1, 21)_ = 6.360, *p* = 0.0198, *η*^2^ = 0.10). No independent or interaction effects of age and activity level were detected on SD1, with no significant difference in either age group or activity level (Figure 5A). There was no significant difference in SD2 between AL-1 and AL-2 in horses under 15 years of age, but this value was lower in older horses who performed AL-1 compared to those who performed AL-2 (61.6 ± 23.3 vs. 86.3 ± 17.7 ms, *p* = 0.0241) (Figure 5B). The SD2/SD1 ratio was significantly lower in horses under 15 years of age who practised AL-1 than those involved in AL-2 (1.5 ± 0.4 vs. 1.8 ± 0.5, *p* = 0.0200), while there was no significant difference between AL-1 and AL-2 in horses 15–20 years old (Figure 5C).

## 4. Discussion

The present study explored how HRV metrics reflect autonomic responses in athletic horses of various ages engaged in typical riding club and equestrian sport activities at varying levels. The key findings of this study are as follows: 1. Interactions between the horses’ age and activity level significantly influenced HRV metrics, particularly pNN50 and DC. Conversely, activity levels independently impacted the expression of the mean HR, mean RR intervals, SDANN, SDNNI, SD2, and the SD2/SD1 ratio. 2. Horses younger than 15 exhibited a lower HR and SD2/SD1 ratios, along with higher RR intervals and pNN50 while training for equestrian dressage compared to those participating in school riding. 3. Horses aged 15 to 20 years demonstrated decreased SDANN, SDNNI, DC, and SD2 during equestrian dressage training relative to their counterparts participating in school riding. These findings indicate that HRV metrics in athletic horses vary based on age and activity level. Horses younger than 15 years displayed heightened HRV during structured training for equestrian sports, whereas those between 15 and 20 years exhibited elevated HRV metrics while engaged in school riding practices.

HRV metrics can be effectively evaluated using 4-, 6-, or 12-lead electrocardiogram (ECG) devices, considered the gold standard in any species [19,34]. However, the complex setup and requirement for multiple optical cables attached to a horse’s body may cause discomfort during physical activity. As a result, HRM devices that detect RR intervals without recording a full ECG have become popular for deriving HRV metrics. These devices offer several advantages over ECG devices, including non-invasiveness, ease of use, lower cost, and greater comfort for the horse [31]. Several HRV metrics derived from HRM devices have been validated [29,30,31,32]. Despite these benefits, the presence of a physiological two-degree atrioventricular (AV) block—common in healthy horses—can lead to missed beats, causing an intermittent prolongation of RR intervals and distortion of HRV results when using HRM devices [35,36]. To mitigate this issue, artefact filtering is necessary to correct for missing beats before conducting HRV analysis [34]. In the current study, automatic artefact filtering was employed to correct or exclude artefacts such as missing beats, extra or misaligned beat detections, ectopic beats, and other arrhythmias. Additionally, the automatic noise detection feature provided by the Kubios programme was utilised to remove noisy segments that could distort the detection of consecutive beats. The implementation of these algorithms aimed to enhance the accuracy of HRV analysis [37]. Consequently, the HRV results from this study are considered reliable for identifying the autonomic responses in horses across varying ages and activity levels.

Horse welfare can be assessed using multiple parameters, including behaviour [38,39], hormonal responses [40], and HRV [28,41]. Studies have shown that increased vagal tone is associated with lower stress levels in mammals [42]. Furthermore, the activities in which horses engage can influence their behavioural and autonomic responses to various challenges, which in turn affects their welfare [43]. Research has also documented that horses recovering from acute abdominal pain exhibit increased mean RR intervals and higher power in the high-frequency (HF) band, indicating elevated vagal activity compared to those on the day of their hospital admission [17]. Therefore, utilising HRV metrics can effectively assess animal welfare in horses. In the present study, HRV metrics varied among different groups of horses and were influenced by age and activity levels. This finding was supported by the interaction observed between age and activity level on the expression of pNN50 and DC, which had a medium effect size. As expected, adult horses undergoing structured dressage training displayed increased RR intervals and pNN50, indicating a decreased HR and a lower SD/SD1 ratio. RR intervals are affected by both sympathetic and vagal activity, and elevated pNN50 signifies a dominance of vagal tone [19,21,44]. A reduced SD2/SD1 ratio also indicates a shift towards vagal activity [20,21]. It is plausible that the superior riding skills of dressage riders, combined with the high exercise capacity of adult horses, could lead to beneficial adaptations and minimise stress for the horses during such training. Our results, therefore, indicate that structured dressage training induced greater vagal activity in adult horses compared to those engaged in a school riding regimen. In contrast, geriatric horses exhibited a decline in SDANN, SDNNI, DC, and SD2. This finding suggests lower HRV, reflecting reduced vagal tone in geriatric horses during structured dressage training compared to their counterparts involved in a school riding regimen. In human studies, measurements of DC, combined with acceleration capacity (AC) and heart rate fragmentation (HRF), have predictive value for cardiovascular disease [45,46]. Low DC and AC combined with a high HRF correlate strongly with cardiovascular issues in patients [45]. In this study, the reduced heart rate variability (DC) observed in geriatric horses raises concerns about the potential negative effects on their autonomic regulation while participating in structured dressage training. Furthermore, the lower DC noted in adult horses compared to geriatric horses involved in a school riding programme also highlights the impact of age on autonomic regulation. The current finding also provides objective evidence to support former anecdotal information from owners; the common perception is that nutrition, grooming, and pain avoidance are of great importance in older horses, while exercise is crucial in younger ones [47]. Accordingly, the current results indicate that adult horses are well-suited for structured training in equestrian sports. In contrast, due to the natural deterioration of their bodily systems, geriatric horses are better suited to lower activity levels, such as those associated with school riding. Therefore, continuing their participation in structured equestrian sport training, which requires high activity levels, may be inappropriate.

Notably, the SDNN measure showed no significant differences between groups of horses of similar ages. However, we observed notable variations in the SDANN and SDNNI metrics among geriatric horses involved in structured dressage training compared to those participating in school riding. This finding is consistent with previous reports from our group, which also indicated significant differences in SDANN and SDNNI but not in SDNN among horses under similar conditions [10,28]. It is likely that fluctuations in RR intervals occur continuously during experiments, even when the horses are at rest. As a result, the overall RR intervals remained relatively stable, leading to a consistent maintenance of SDNN throughout the 30 min intervals measured. However, significant differences emerged when variation was calculated over consecutive 5 min segments within that 30 min duration. This indicates that evaluating these 5 min segments in terms of SDANN and SDNNI provides a more detailed analysis of the standard deviation of RR intervals, offering advantages over using SDNN alone during the experiment. Therefore, assessing HRV metrics over consecutive 5 min intervals may be more sensitive and should be the preferred method for indicating HRV modulation rather than using longer recording periods. The expression of resting HR and autonomic responses varied among horses of different ages and activity levels in this study. However, there remains a gap in our understanding of how autonomic regulation manifests during exercise regimens, equestrian competitions, and recovery in horses under these conditions.

Several limitations of this experiment must be considered. While there was a structured timeline for the exercise training programmes focused on equestrian dressage and school riding, the order of the exercises—such as trotting or cantering—was not standardised. This introduced variability dependent on the preferences of each horse’s trainer. Moreover, individual differences in behavioural and HRV parameters persist among horses [48,49,50]. The experiment involved a large number of horses and included the recording of consecutive RR intervals and echocardiography. However, each horse unavoidably underwent examination at different times of the day, and circadian rhythm may also affect the expression of HRV metrics [51,52]. These factors could have contributed to within-group variations when HRV metrics were evaluated in this study. Therefore, the results should be interpreted with caution.

## 5. Conclusions

Horses under 15 years of age demonstrate better autonomic regulation when trained for equestrian sports compared to those involved in a school riding programme. Conversely, continuous training for equestrian sports in horses aged 15 to 20 years tends to reduce autonomic regulation in comparison to the school riding regimen. Therefore, caution should be exercised when continuing structured equestrian sports training for horses older than 15 years, as it may cause increased stress during training in advanced age, potentially compromising horse welfare. This study provides insights into how autonomic responses vary in horses based on age and activity levels. These findings have significant implications for horse welfare, highlighting the importance of careful management to ensure that horses, particularly older ones, participate in appropriate levels and types of physical activity to safeguard their well-being.

## Figures and Tables

**Table 1:** Categories of horses with different ages and activity levels.

Age Ranges	Age Groups (Years) (Mean ± SD)	Activity Levels	
<15 years	10.8 ± 2.9 (N = 13)	AL-1	-
	10.4 ± 2.8 (N = 13)	-	AL-2
15–20 years	17.5 ± 2.3 (N = 10)	AL-1	-
	18.2 ± 1.9 (N = 10)	-	AL-2

## Data Availability

Data supporting this study are available at https://www.doi.org/10.6084/m9.figshare.28832186 (accessed on 22 April 2025).

## Abbreviations

The following abbreviations are used in this manuscript:

AL-1	structured exercise programme for equestrian dressage practise three to five days/week
AL-2	school riding practice one or less than one day/week
ANOVA	analysis of variance
CSV	comma-separated values
DC	deceleration capacity of heart rate computed as a four-point difference
HF	high-frequency band
HR	heart rate
HRM	heart rate monitoring
HRV	heart rate variability
LF	low-frequency band
LSD	least significant difference
pNN50	number of successive RR interval pairs that differ more than 50 ms divided by the total number of RR intervals
RMSSD	square root of the mean squared differences between successive RR intervals
RR	beat-to-beat
SDNN	standard deviation of normal-to-normal RR intervals
SDANN	standard deviation of the averages of normal-to-normal RR intervals in 5 min segments
SDNNI	mean of the standard deviations of normal-to-normal RR intervals in 5 min segments
SD1	standard deviation in Poincaré plot perpendicular to the line of identity
SD2	standard deviation in Poincaré plot along the line of identity
